# Elastase-Activated Antimicrobial Peptide for a Safer Pulmonary Treatment of Cystic Fibrosis Infections

**DOI:** 10.3390/antibiotics11030319

**Published:** 2022-02-28

**Authors:** Margherita Degasperi, Riccardo Sgarra, Mario Mardirossian, Sabrina Pacor, Massimo Maschio, Marco Scocchi

**Affiliations:** 1 Department of Life Sciences, University of Trieste, 34127 Trieste, Italy; margherita.degasperi@areasciencepark.it (M.D.); rsgarra@units.it (R.S.); mmardirossian@units.it (M.M.); pacorsab@units.it (S.P.); 2 ARGO Open Lab Platform for Genome Sequencing, AREA Science Park, Padriciano, 34149 Trieste, Italy; 3 Institute for Maternal and Child Health, IRCCS Burlo Garofolo, 34134 Trieste, Italy; massimo.maschio@burlo.trieste.it

**Keywords:** antimicrobial peptide, cystic fibrosis, pro-drug, elastase, *Pseudomonas aeruginosa*

## Abstract

As bioactive small proteins with antimicrobial and immunomodulatory activities that are naturally produced by all living organisms, antimicrobial peptides (AMPs) have a marked potential as next-generation antibiotics. However, their development as antibacterial agents is limited by low stability and cytotoxicity. D-BMAP18, a membrane-permeabilizing antimicrobial peptide composed of D-amino acids, has shown good antibacterial and anti-inflammatory activities but also a non-negligible cytotoxicity against eukaryotic cell lines. In this study, a prodrug has been developed that extends the peptide with a negatively charged, inactivating sequence containing the cleavage site for neutrophil elastase (NE). The ultimate goal was to allow the activation of D-BMAP18 by endogenous elastase only at the site of infection/inflammation, enabling a slow and targeted release of the pharmacologically active peptide. In vitro activation of Pro-D-BMAP18 was confirmed using purified NE. Its antimicrobial and cytotoxic activities were tested in the presence and absence of elastase and compared to those of the parental form. The prodrug had minimal activity in the absence of elastase, while its proteolysis product retained an appreciable antimicrobial activity but lower cytotoxicity. Moreover, Pro-D-BMAP18 was found to be correctly converted to D-BMAP18 in the presence of CF sputum as a model of the lung environment and showed good antimicrobial activity under these conditions.

## 1. Introduction

The morbidity and mortality of cystic fibrosis patients (CF) is mostly caused by respiratory failure due to chronic lung infections [1]. Intensive antibiotic therapy for bacterial lung infections contributes to the development of multidrug-resistant (MDR) pathogens [2]. In addition, the spread of pathogens in the form of biofilm communities, which are naturally resistant to many antibiotics, makes it difficult to eradicate infections [3,4]. *Pseudomonas aeruginosa* is the most commonly described opportunistic pathogen affecting CF patients [5]. In addition, several studies have reported that lung function declines more rapidly in patients chronically coinfected with *Candida* spp. and *P. aeruginosa* [6].

Antimicrobial resistance requires the development of novel antimicrobial agents effective against multidrug-resistant strains to complement or replace current antibiotic therapies. In this scenario, antimicrobial peptides (AMPs) are interesting molecules due to their broad spectrum of activity against MDR strains [7,8,9]. Antimicrobial peptides (AMPs) are key components of the innate immune system of vertebrates and invertebrates, where they exert multiple functions, including direct antimicrobial activity and immunomodulatory activities that stimulate the immune system and suppress the inflammatory response [9,10,11,12,13].

The killing mechanism of AMPs is mainly based on permeabilization of the microbial membrane [11,14], although other mechanisms based on multiple molecular targets may accompany or replace membrane permeabilization [15]. AMPs have often demonstrated the ability to circumvent common microbial resistance mechanisms to antibiotics [10], a property likely related to their multiple killing mechanisms and lack of unique molecular targets [11,12] and to their synergistic activity between AMPs and antibiotics [16].

The therapeutic potential of various AMPs against CF isolates has been widely reported [17,18,19,20]. Some of them, the cathelicidins, have also been tested against CF isolates [21,22] and shown potent broad-spectrum antimicrobial activity in vitro against Gram-positive and Gram-negative bacteria and fungi, although they were quite cytotoxic to human erythrocytes and neutrophils at higher concentrations relative to microbicidal ones [23]. A truncated form of cathelicidin BMAP-27, termed BMAP-18, which lacks nine C-terminal hydrophobic residues, showed antimicrobial activity against the planktonic form of *P. aeruginosa* CF isolates [24], with efficacy comparable to tobramycin and in some cases even higher [22,24]. Unfortunately, the peptide was ineffective against acute lung infections in mice, probably because of its rapid degradation [24]. Its enantiomeric form, D-BMAP18, showed non-negligible cytotoxicity against different cell lines [25,26] despite its high antibacterial and anti-inflammatory properties also in the presence of CF sputum [25].

A prodrug approach has been used previously to reduce toxicity and increase specificity through selective activation [27]. Forde and colleagues designed several AMP prodrugs such that their net positive charge was masked by a negative pro-moiety containing a substrate for the enzyme neutrophil elastase (NE). These prodrugs of AMPs limited the cytotoxic effects of AMP treatment and made antimicrobial activity dependent on the host enzyme NE. The pro-AMP modification reduced host toxicity in a mouse model of lung administration, with the pro-peptide being less toxic than the active peptide [28]. However, the results depended on the type of peptide used, and there is still considerable scope for improving the selectivity of pro-AMPs [28].

Mammals produce cathelicidins as inactive proforms which are processed by NE to obtain active peptides [29,30]. The aim of this study was to reproduce this activation process by designing a BMAP-18 proform that is activated by NE only at the site of infection/inflammation, reducing its residual cytotoxicity. Pro-D-BMAP18 was synthesized, its proteolytic activation was tested, and its antimicrobial activity and cytotoxicity before and after proteolytic activation were investigated. The results provided new insights into the biocompatibility of AMPs for the treatment of lung infections in CF.

## 2. Results

### 2.1. Design of the Pro-Peptide Pro-D-BMAP18 Activated by the Proteases in the Site of Infection

**Figure 1:**
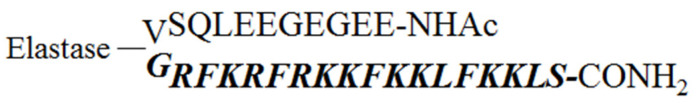
Scheme and sequence of Pro-D-BMAP18. The mature D-peptide released upon cleavage is shown in italics and bold. The line represents the neutrophil elastase cleavage site. The turn encompassing Val11 and Gly12 was predicted using the PepFold3 tool.

To obtain a stable inactive form of D-BMAP18 that releases the active peptide at the site of infection by specific proteolysis, a pro-drug peptide was designed and synthesized. This peptide was designed with a short, negatively charged N-terminal pro-region linked to a cationic C-terminal part corresponding to the D-BMAP18 peptide. The chimeric peptide contained a cleavage site for elastase between the two parts to allow the release of the active D-BMAP18. The C-terminus and the N-terminus of the pro-peptide were amidated and acetylated, respectively, to remove any negative charge in the active C-terminal peptide and any positive charge in the N-terminal pro-form (Figure 1). In this way, the endogenous elastase present at the site of infection/inflammation might be able to process the pro-peptide in situ and minimize its cytotoxicity.

### 2.2. Antimicrobial Activity of Pro-D-BMAP18 against Bacterial and Fungal Strains

To determine whether the pro-sequence of Pro-D-BMAP18 effectively sequesters the active peptide impairing its activity, the antimicrobial activity of Pro-D-BMAP18 and D-BMAP18 were tested against 12 different *P. aeruginosa* isolates from CF patients which have been previously characterized [22]. The Pro-D-BMAP18 displayed a dramatic decrease (4–32 fold) of its antimicrobial activity compared to free D-BMAP18, confirming that the pro-sequence efficiently impaired the antimicrobial activity of D-BMAP18 (Table 1). 

### 2.3. Proteolytic Activation of Pro-D-BMAP18

To assess whether the pro-peptide could release the active D-BMAP18 after elastase digestion, cleavage of Pro-D-BMAP18 was evaluated in vitro by incubating the pro-drug with neutrophil elastase (NE). After 1 h incubation, 50% of the prodrug was cleaved, and the cleavage was complete after 4 h incubation. Mass spectrometry analysis after cleavage identified only D-BMAP18 (Mw = 2342). No other products were detected, demonstrating the specificity of the cleavage and that no other unwanted molecules were released during proteolysis (Figure S1).

### 2.4. Antimicrobial Activity of Pro-D-BMAP18 in the Presence of Elastase and in the Supernatant of HL-60 Cells

The antimicrobial activity of the peptide released from the pro-drug by proteolysis was evaluated by MIC assays, in which the Pro-D-BMAP18 was incubated with two different strains of *P. aeruginosa* (PAO1 and RP37) and two strains of *C. albicans* (ATCC 90029 and SC 5314) in the presence of NE.

Recovery of antibacterial activity of the released D-BMAP18 was clear but incomplete compared to free D-BMAP18. The released peptide recovered its full antimicrobial activity only after the addition of 300 mM NaCl to the medium (final concentration = 450 mM) (Table 2). The activity of cleaved Pro-D-BMAP18 was also evaluated against the synthesis of new biofilm at sub-MIC concentrations and against preformed biofilm. The pro-peptide incubated in the presence of NE inhibited the formation of new biofilm with activity comparable to the parental form (Figure S2).

The release of the active D-BMAP18 moiety was also evaluated by incubating the Pro-D-BAMP18 with the exhausted medium of degranulated neutrophil-like cells which had been differentiated from HL-60 cells and therefore enriched in proteases. Pro-D-BMAP18 was incubated for various times with this conditioned medium and each sample was used to perform an MIC assay against *P. aeruginosa* cells. The results showed a time dependent conversion of the pro-peptide to D-BMAP18, suggesting that neutrophils released sufficient amount of elastase to process Pro-D-BMAP18 into D-BMAP18 (Table 2).

### 2.5. Biocompatibility of the Pro-D-BMAP18 in the Presence of Elastase

**Figure 2:**
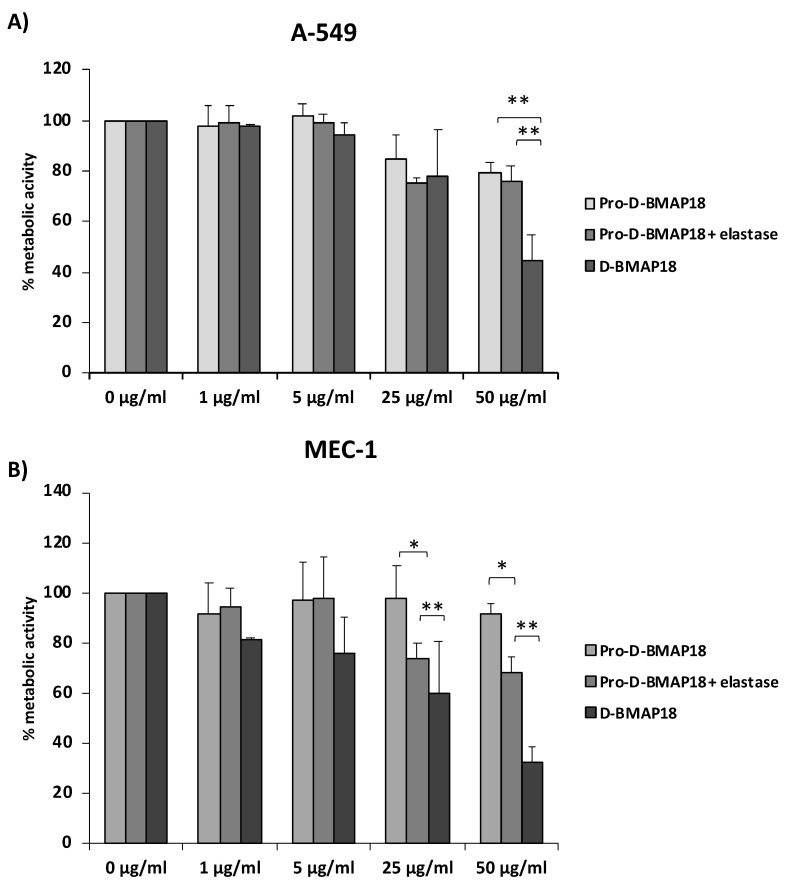
Cell viability assay in the presence of NE-activated Pro-D-BMAP18. An MTT assay was performed using (**A**) adenocarcinomic human alveolar basal epithelial cells A-549 and (**B**) B-chronic lymphocytic leukaemia cells MEC-1. The percentages shown are of metabolically active cells compared with untreated controls after 24 h incubation with different concentrations of Pro-D-BMAP18 with or without NE and D-BMAP18. Results of three different experiments in internal triplicate (n = 9). * = *p* < 0.05; ** = *p* < 0.01. A Student’s n = of the treatments vs. the untreated control is reported.

To evaluate its biocompatibility, Pro-D-BMAP18 and human NE were incubated with the adenocarcinomic human alveolar basal epithelial cells A-549 or chronic lymphocytic leukaemia cells MEC-1 for 24 h. Free D-BMAP18 was used in parallel as a comparison. Compared with the untreated control, 50 μg/mL Pro-D-BMAP18 in the presence of elastase decreased the viability of A-549 cells by 20%. A similar result was observed in the absence of elastase. In contrast, D-BMAP18 at the same concentration reduced viability by 60% (Figure 2).

Treatment of MEC-1 cells with Pro-D-BMAP18 and NE at a concentration of 50 μg/mL reduced cell viability by less than 30% compared with the untreated control, whereas D-BMAP18 at the same concentration reduced cell viability by 70% compared with the same control. A higher viability for NE-treated Pro-D-BMAP18 than for D-BMAP18 was also observed at 25 µg/mL (Figure 2).

Overall, these data indicate very low cytotoxicity for Pro-D-BMAP18, which increased weakly in the presence of elastase and remained lower than that for D-BMAP18, suggesting that gradual activation of the pro-form within 24 h leads to a significant reduction in cytotoxicity.

### 2.6. Antimicrobial Activity of Pro-D-BMAP18 in CF Sputum against P. aeruginosa RP73 or C. albicans SC 5314

**Figure 3:**
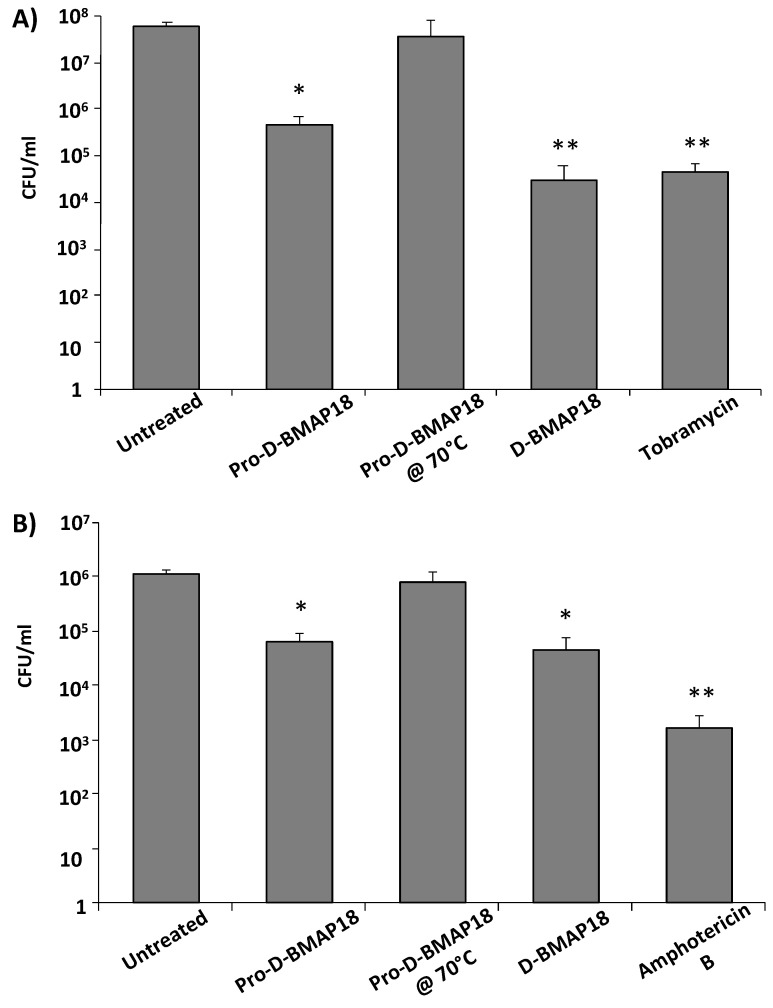
Antimicrobial activity of Pro-D-BMAP18 in 25% (*v*/*v*) CF sputum in SCFM + 300 mM NaCl. CFU/mL count of (**A**) *P. aeruginosa* RP73 and (**B**) *C. albicans* SC5314 after 4 h incubation with Pro-D-BMAP18 (64 μg/mL), D-BMAP18 (64 μg/mL), tobramycin (8 μg/mL), and amphotericin B (1 μg/mL). Pro-D-BMAP18 (64 μg/mL) in heat-inactivated sputum was used as a negative control for enzymatic activity. Results are from three separate experiments in internal duplicate (n = 6). * = *p* < 0.05; ** = *p* < 0.01. A Student’s *t*-test of the treatments vs the untreated control is indicated.

Finally, the antimicrobial activity of Pro-D-BMAP18 was tested in CF sputum. It was investigated whether the proteolytic activity of endogenous elastase in CF sputum was sufficient to process Pro-D-BMAP18 and unmask its antibacterial activity. The number of viable CFUs of the *P. aeruginosa* strain RP73 and *C. albicans* strain SC5314 was determined by incubating the pathogens with Pro-D-BMAP18 in 25% (*v*/*v*) CF sputum diluted in SCFM with 300 mM NaCl added, a medium that mimics the chemical composition of CF sputum [22]. Pro-D-BMAP18 acquired antimicrobial activity in CF sputum. After 4 h incubation the bactericidal efficacy of Pro-D-BMAP18 was comparable to that of the D-BMAP18, displaying a 2-log reduction of viable *P. aeruginosa* RP73 cells and an approximately 1-log reduction of *C. albicans* cells (Figure 3). When the pro-drug was incubated with CF sputum samples that had been heat-inactivated to kill any enzymatic activity, the peptide did not recover its antimicrobial activity. This confirmed the role of the elastase (or other unspecified proteolytic enzymes in CF sputum) in cleaving Pro-D-BMAP18 to its active form, D-BMAP18. Thus, the sputum from CF patients contained sufficient active proteases (elastase) to convert the Pro-D-BMAP18 into a compound (D-BMAP18), exerting antimicrobial activity under the physio(patho)logical conditions of CF lungs.

## 3. Discussion

Pro-D-BMAP18, an inactive form of D-BMAP18, was developed as a safer antimicrobial compound for the treatment of lung infections. This involved modifications that have been used for other antibacterial agents [27,31,32]. Neutrophils represent the major cell population among the inflammatory cells recruited to the airways of CF patients, where they are responsible for the in situ release of elastase [33]. For this reason, Pro-D-BMAP18 was developed by adding an inactivating extra sequence to the N-terminus of D-BMAP18, which contains the cleavage site for elastase. The presence of this specific cleavage site should allow the gradual release of the active antimicrobial peptide only in environments enriched with NE, such as the inflamed and infected CF lung. This rationale had already been used by Forde and colleagues [20,27] to convert other antimicrobial peptides into safer pro-drugs in both in vitro and in vivo experiments [20]. Differently to them, we designed the pro-drug using an optimized cathelicidin as the active antimicrobial component and we introduced the native cleavage site for elastases present in the cathelicidin sequence to exploit this property.

The pro-sequence successfully blocked the peptide and prevented its antimicrobial effect as well as its undesirable cytotoxicity toward eukaryotic cells. The pro-peptide was then correctly converted into D-BMAP18 by elastase, demonstrating that Pro-D-BMAP18 was activated only under the conditions for which it was designed. However, the electrostatic interaction between the anionic pro-sequence and the cationic active peptide probably kept the two moieties bound to each other in part of the molecular population even after cleavage. This eroded some of the antimicrobial activity of the pro-drug compared with native D-BMAP18, as the released peptide fully recovered its antimicrobial activity only in the presence of 450 mM NaCl when tested in CF mucus against both bacterial and fungal strains. Most likely, the addition of salts also displaces the remaining pro-sequences from active D-BMAP18, unmasking its full antimicrobial activity, and might help to avoid excessive interaction with mucus. However, the presence of NaCl does not represent a major problem for potential therapeutic applications of Pro-D-BMAP18, as the compound per se shows high antimicrobial activity both in the presence and absence of high NaCl concentrations. In contrast, our data raise the interesting possibility of combining the use of Pro-D-BMAP18 with sodium chloride. Aerosol administration of hypertonic NaCl solutions is already used to treat CF patients to soften mucus [34]. Therefore, Pro-D-BMAP18 could be administered in these treatments not only without interference but even with enhancement of its antimicrobial activity.

To be sure that Pro-D-BMAP18 can be used under the really difficult conditions of CF sputum, we demonstrated that the number of neutrophil elastases or other proteolytic enzymes present in this CF sputum was sufficient to convert Pro-D-BMAP18 to D-BMAP18 within a few hours. This was previously observed by Forde and colleagues [27], but we have expanded the group of pathogens tested to include *C. albicans*, which frequently co-infects the lungs of CF patients. In contrast to Forde, who reported a 20% reduction in bacterial load after pro-drug treatment in CF human bronchoalveolar lavage, we observed a 2-log reduction by treatment with Pro-D-BMAP18 activated in diluted CF sputum. Thus, the in situ release of D-BMAP18 from the pro-drug could proceed correctly in the lungs of patients thanks to the endogenous enzymes that are always present in inflamed lungs [35]. 

Interestingly, activated pro-D-BMAP18 has a much smaller effect on human cell lines in vitro than D-BMAP18. This suggests that the slow elastase-dependent release of D-BMAP18 from Pro-D-BMAP18 reduces the deleterious effects of administering high doses of active peptides to host cells. It is therefore tempting to also assume a higher in vivo biocompatibility of Pro-D-BMAP18 compared to D-BMAP18, which has shown significant side effects both in cell cultures and in vivo [26]. Further experiments in this direction are planned. The demonstrated improvement in biocompatibility of Pro-D-BMAP18, at least in vitro, is relevant to the scenario of the clinical application of AMPs, since one of the most common side effects of AMPs is acute toxicity in vitro and in vivo [36,37]. All these observations confirmed that in situ activation of Pro-D-BMAP18 can overcome the side effects of D-BMAP18 and that it exhibits lower in vitro cytotoxicity and retains the antimicrobial properties of the parental form. These results will direct evaluation of the in vivo effects of the pro-drug to determine whether it can be introduced into the lungs of infected animals. Peptide antibacterial pro-drugs were recently administered into the lungs using a vibrating mesh nebulizer, suggesting that the pro-drug modification is not harmful [20] and supporting further development of pro-AMPs as therapeutics in CF.

In conclusion, to date, more than 3000 AMPs have been discovered, but only seven of them have been approved by the FDA due to issues related to stability and/or toxicity [37]. We have shown that the addition of a specific, inactivating extra sequence to an antimicrobial D-peptide could be a promising solution for the specific in situ activation of AMPs. By adding a negatively charged extra sequence to D-BMAP18, we could obtain a safe Pro-D-BMAP18 peptide that retains the antimicrobial activity of the parental form but has higher biocompatibility towards human cells. This type of formulation could be easily applied to other cathelicidins and could be a promising solution for the in vivo clinical application of antimicrobial peptides.

## 4. Materials and Methods

### 4.1. Bacterial and Fungal Strains

Previously characterized *Pseudomonas aeruginosa* strains, isolated from CF patients, were used. Each CF isolate was resistant to at least three of the following groups of antibiotics: β-lactams with or without β-lactamase inhibitor, aminoglycosides, fluoroquinolones, folate-pathway inhibitors (trimethoprim-sulphamethoxazole), tetracyclines, and macrolides. *P. aeruginosa* RP73, and *P. aeruginosa* PAO1 were used as reference strains [21,22]. All strains were stored at −80 °C until use and plated on Mueller–Hinton agar (MHA; Oxoid S.p.A., Milan, Italy). The *Candida albicans* strains used were ATCC 90029 and SC 5314. All strains were stored at −80 °C until used and plated on Sabouraud agar (Sigma-Aldirch, MO, USA). 

### 4.2. Antimicrobial Compounds and Synthesis of Pro-D-BMAP18

D-BMAP18 (GRFKRFRKKFKKLFKKLS-am) (≥95% purity) was purchased from NovoPro Bioscience Inc. (Shangai, China), freeze-dried three times from HCl 10 mM to remove residual TFA (CT60e Heto, Technology of Scandinavia) and finally resuspended in sterile H_2_O. Tobramycin was purchased from Sigma-Aldrich (St. Louis, MO, USA) and resuspended in H_2_O at a final concentration of 5 mg/mL. Amphotericin b was purchased from Sigma-Aldrich (St. Louis, MO, USA) and resuspended in H_2_O at a final concentration of 10 mg/mL. All peptides and antibiotics were stored at −20 °C. 

Pro-D-BMAP18 (Ac-EEGEGEELQSV*GRFKRFRKKFKKLFKKLS*-am; D-residues are reported in bold and italics) was synthesized according to Mardirossian and colleagues [26]. Before cleaving the pro-peptide from the resin, its N-terminus was acetylated incubating the resin for 5 min in 20% (*v*/*v*) acetic anhydride in dimethylformamide at room temperature. Subsequently, 1.5 equivalent of N,N-Diisopropylethylamine (DIPEA) was added to the solution, prolonging the incubation for another 30 min. The resin was then washed twice with dimethylformamide, dichloromethane, and methanol. The efficiency of the acetylation reaction was checked using a Keiser test kit (Sigma-Aldrich), according to the instructions of the supplier. All of these reagents were purchased from Sigma-Aldrich. The peptide was then cleaved from the resin as reported in [26], lyophilized three times with 10 mM HCl (CT60e Heto, Technology of Scandinavia) to remove residual TFA, and resuspended in sterile H_2_O. 

The concentration of the stock solution of peptides was evaluated by spectrophotometric determination of peptide bonds (ε_214nm_) according to Kuipers and Gruppen, with slight modifications [38].

### 4.3. Cleavage of Pro-D-BMAP18 by Human Elastase in HEPES and by HL-60 Cells

Pro-D-BMAP18 (500 ng) was mixed with human neutrophil elastase (Human Neutrophil Elastase, Abcam, Cambridge, UK) at a molar ratio of 100:1, respectively, and incubated at 37 °C in 50 μL HEPES 100 mM pH 7.0. Then, 15 μL of the mixture were withdrawn at different time points, the reaction was stopped by dilution in 15 μL of ice-cold H_2_O + 0.1% (*v*/*v*) TFA and frozen at −20 °C until HPLC-MS analysis. Each sample was analysed by HPLC using the analytic column Symmetry^®^ C18 (100 Å, 3 μm, 3 mm × 100 mm; Waters, Billerica, MA, USA). The elution was performed by a linear gradient of solvent B (0.05% *v*/*v* TFA in AcCN) in A (0.05% *v*/*v* TFA in water) from the 20% (*v*/*v*) to the 35% (*v*/*v*) of B in 15′ under a flow rate of about 15 μL/min. The HPLC was connected to the mass analyser HCT ultra (Bruker Daltonics, Billerica, MA, USA) equipped with an electrospray ionization (ESI-MS) system and using a capillary voltage of 200 V.

### 4.4. Preparation of Conditioned Medium from Neutrophil-like Cells 

The promyelocytic human cells HL-60 (ATCC CCL-240™) were grown in RPMI medium (Sigma-Aldrich, St. Louis, MO, USA) supplemented with 20% (*v*/*v*) fetal bovine serum (FBS, EuroClone, Milan, Italy), 100 U/mL penicillin/streptomycin (Sigma-Aldrich, MO, USA) and 2 mM L-glutamine (EuroClone, Milan, Italy) at 37 °C in 5% CO_2_ in 96-well U-bottom microtiter plates (Sarstedt, Milan, Italy). Differentiation to neutrophil-like cells was induced by culturing the cells in the presence of 100 nM phorbol myristate acetate (PMA, Sigma-Aldrich, St. Louis, MO, USA) for 6 days [39]. Differentiation was assessed by cell cycle analysis. Then, 10^6^ cells were fixed in 1 mL ethanol and washed twice using 1 mL PBS (Sigma-Aldrich, MO, USA, pH = 7.4) for 5 min at 500× *g*. Cells were stained with a solution of 1% (*v*/*v*) propidium iodide (PI; Sigma-Aldrich, St. Louis, MO, USA, 1×), 0.5% (*v*/*v*) FITC (Sigma-Aldrich, MO, USA), and 0.4% (*v*/*v*) RNasi A (Sigma-Aldrich, MO, USA) in PBS. After overnight incubation at 4 °C, cells were analysed by the cytofluorimeter Cytomics FC 500 (Beckman-Coulter, Inc., Fullerton, CA, USA). To induce the release of elastase by the differentiated HL-60 cells, after 6 days of incubation, the exhausted medium was discarded and 1 mL of fresh medium containing 100 μM PMA (Sigma-Aldrich, St. Louis, MO, USA) was added to the wells for 2 h. The medium and the cells were collected and centrifuged at 300× *g* for 10 min. The supernatant was collected for further assays.

### 4.5. Evaluation of Antimicrobial Activity

The antibacterial activity of the peptides was evaluated as MIC (minimum inhibitory concentration) in both the MH and supernatant of HL-60 cells. Two-fold serial dilutions of Pro-D-BMAP18 (128 μg/mL) in the presence of elastase (12.8 μg/mL) at 100:1 molar ratio and D-BMAP18 were prepared in MH medium in 96-well U-bottom microtiter plates (Sarstedt, Milan, Italy) following the EUCAST guidelines. Briefly, serial two-fold dilutions of the compounds were prepared in the medium and aliquoted in round-bottom 96-well microtiter plates (Sarstedt). Each well was inoculated with a standardized inoculum, grown overnight, then re-grown in new medium to optical density at 600 nm ≈ 0.3, and diluted to achieve a final test concentration of about 2.5 × 10^5^ CFU/mL. The MIC was measured as the lowest concentration of the peptide that completely inhibited visible bacterial growth after incubation at 37 °C for 24 h.

Concerning the antimicrobial activity in the exhausted medium of differentiated HL-60 cells, Pro-D-BMAP18 (final concentration: 128 μg/mL) was incubated in the supernatant for 4 h, 18 h and 24 h. For each time-point, 100 μL of the medium containing the pro-peptide was taken and used for an MIC assay against the *P. aeruginosa* strains PA01 and RP73, as reported above.

The antifungal activity (MIC) against both the *C. albicans* strains ATCC90029 and SC 5314 was tested in Sabouraud broth (Sigma-Aldirch, MO, USA) according to the guidelines of the National Committee for Clinical Laboratory Standards. Fungal colonies were resuspended in 5 mL sterile PBS and the optical density (OD) was measured at 600 nm (Ultraspec 2100 pro, Amersham Bioscience, Buckinghamshire, UK). Each well was inoculated with a standardized inoculum of 50 μL to achieve a final test concentration of 5 × 10^4^ CFU/mL. After incubation of the plate at 30 °C for 48 h, MIC was determined as the lowest concentration of antimicrobial peptide resulting in the complete inhibition of visible growth.

The biofilm eradication protocol (see Figure S1) was performed according to [22] for the cultivation of bacterial and biofilm and using the protocol described in [22] for its staining with MTT.

### 4.6. In Vitro Cytoxicity Assays

Cytotoxicity was evaluated as cell viability by the MTT assay using two different human cells lines: the adenocarcinomatous human alveolar basal epithelial cells A-549 and the human lymphoid MEC-1 cells (DSMZ, ACC 497). A-549 and MEC-1 cells grew in Dulbecco’s MEM (Sigma, Milan, Italy) added in 10% (*v*/*v*) FBS (EuroClone, Milan, Italy), 100 U/mL penicillin/streptomycin (Sigma-Aldrich, MO, USA), and 2 mM L-glutamine (EuroClone, Milan, Italy) at 37 °C in 5% (*v*/*v*) CO_2_. Then, 20,000 cells/50 μL were seeded in each well of a 96-well flat-bottom microtiter plate (Sarstedt, Milan, Italy) and incubated overnight at 37 °C in 5% CO_2_. Serial twofold dilutions of D-BMAP18, Pro-D-BMAP18, and Pro-D-BMAP18 in the presence of elastase (molar ratio, 100:1) were prepared in the same cell growth medium, and 50 μL of the samples were added to the cells. After 20 h of incubation at 37 °C under 5% (*v*/*v*) CO_2_, 20 μL of MTT (5 mg/mL in PBS) was added to each well. After 4 h incubation at 37 °C in the dark, 100 μL of 10% (*w*/*v*) Igepal (Sigma-Aldrich, MO, USA) in 0.01N HCl was added to each well and the plate was incubated overnight at 37 °C under 5% (*v*/*v*) CO_2_. Cell viability was evaluated as absorbance at 570 nm using the Nanoquant infinite M200pro multiplate reader (Tecan, Mannedorf, Switzerland).

### 4.7. Bacterial Killing Assay in CF Sputum

Evaluation of antibacterial activity in the sputum of CF patients was measured as bacterial kill assay against the *P. aeruginosa* strain RP73.

Sputum samples were collected and pooled as already described [25] The pool was then divided into aliquots and frozen at −20 °C. The assay was performed in 25% (*v*/*v*) sputum in SCFM (synthetic cystic fibrosis sputum medium [22,36]) with the addition of NaCl to a final concentration of 450 mM. Different concentrations of the peptide were mixed with 100 μL of diluted sputum for 1h at 37 °C in a thermostatically controlled water bath with 100 μL of bacterial cells (10^6^ CFU/mL) resuspended in 25% (*v*/*v*) sputum in SCFM. After incubation, tenfold dilutions of each sample were prepared in MH broth (Oxoid S.p.A., Milan, Italy) and 25 μL of each solution were plated on MH agar plates (Oxoid S.p.A., Milan, Italy) and incubated overnight for further colony counting.

For *C. albicans* SC 5314, the assay was performed as described above, but the concentration of fungal cells was approximately 5 × 10^4^ CFU/mL and the medium used for dilution and plating was Sabouraud (Sigma-Aldirch, MO, USA) with and without agar, respectively.

In both cases, a plate containing a serial dilution of 25% (*v*/*v*) CF sputum alone without the addition of bacteria/fungi was used as a control for the presence of endogenous bacterial flora. No endogenous bacterial growth was observed in the control plate after incubation due to the different growth speeds of the endogenous strains.

### 4.8. Statistical Analysis

For statistical analysis, data from at least three independent experiments were used, which were internal triplicate experiments. Differences between groups were evaluated using the unpaired Student’s *t*-test (biofilm formation) or the ANOVA test (cytotoxicity).

## Figures and Tables

**Table 1:** MIC (μg/mL) values of D-BMAP18 vs. Pro-D-BMAP18 against *P. aeruginosa* strains.

Strains	Pro-D-BMAP18	D-BMAP18
PAO1	128	4
RP 73	128	4
PA 03	128	8
PA 05	32	4
PA07	64	8
PA08	128	8
PA 09	64	4
PA 10	64	8
PA 14	64	16
PA 21	128	16
PA 22	16	4
PA 31	128	16

**Table 2:** MIC values (μg/mL) of D-BMAP18 and of D-BMAP18 released from Pro-D-BMAP18 incubated with elastase or elastase-containing conditioned cell medium.

Compound	*P. aeruginosa*		*C. albicans*	
	PAO1	RP73	ATCC 90029	SC5314
D-BMAP18	16	8	2	16
Pro-D-BMAP18	128	128	16	>128
Pro-D-BMAP18 + NE	32	32	4	32
Neutrophil elastase (NE)	>128	>128	>128	>128
Pro-D-BMAP18 + NE + 350 mM NaCl	16	8	2	16
Pro-D-BMAP18 + cell supernatant, 4 h	64	64	nd	nd
Pro-D-BMAP18 + cell supernatant, 18 h	32	32	nd	nd
Pro-D-BMAP18 + cell supernatant, 24 h	32	32	nd	nd

The results are the mode of three independent experiments performed in duplicate. nd: not determined.

## Data Availability

Although not involving any procedure on patients, since the sputum was waste material autonomously expelled by patients and no data on patients were available, informed consent was obtained from all subjects involved in the study.

## Supplementary Materials

The following supporting information can be downloaded at: https://www.mdpi.com/article/10.3390/antibiotics11030319/s1, Figure S1: Proteolytic cleavage of Pro-D-BMAP18, Figure S2: Anti-biofilm activity of Pro-D-BMAP18.
