# Supplementary material for: Elastase-Activated Antimicrobial Peptide for a Safer Pulmonary Treatment of Cystic Fibrosis Infections
Source: Antibiotics (Basel). 2022 Feb 28;11(3):319. doi: 10.3390/antibiotics11030319 (PMC8944445; doi:10.3390/antibiotics11030319)
Supplement: Supplementary file 1 [file antibiotics-11-00319-s001.zip › antibiotics-1560323-supplementary.pdf]

antibiotics

MDPI

Supplementary Materials

Article

Elastase-Activated Antimicrobial Peptide for a Safer Pulmonary Treatment of Cystic Fibrosis Infections

Margherita Degasperi [1,2], Riccardo Sgarra [1], Mario Mardirossian [1], Sabrina Pacor [1], Massimo Maschio [3] and Marco Scocchi [1,*]

[1] Department of Life Sciences, University of Trieste, 34127 Trieste, Italy; margherita.degasperi@areasciencepark.it (M.D.); rsgarra@units.it (R.S.); mmardirossian@units.it (M.M.); pacorsab@units.it (S.P.)
[2] ARGO Open Lab Platform for Genome Sequencing, AREA Science Park, Padriciano, 34149 Trieste, Italy
[3] Institute for Maternal and Child Health, IRCCS Burlo Garofolo, 34134 Trieste, Italy; massimo.maschio@burlo.trieste.it
* Correspondence: mscocchi@units.it

Mass spectrometry analysis of Pro-D-BMAP18 after cleavage by neutrophil elastase (NE) identified only D-BMAP18 (Mw=2342). 50% of the prodrug was cleaved after 1h incubation and the cleavage was complete after 4h incubation. No other products were detected, demonstrating the specificity of the cleavage and that no other unwanted molecules were released during proteolysis (Figure S1).

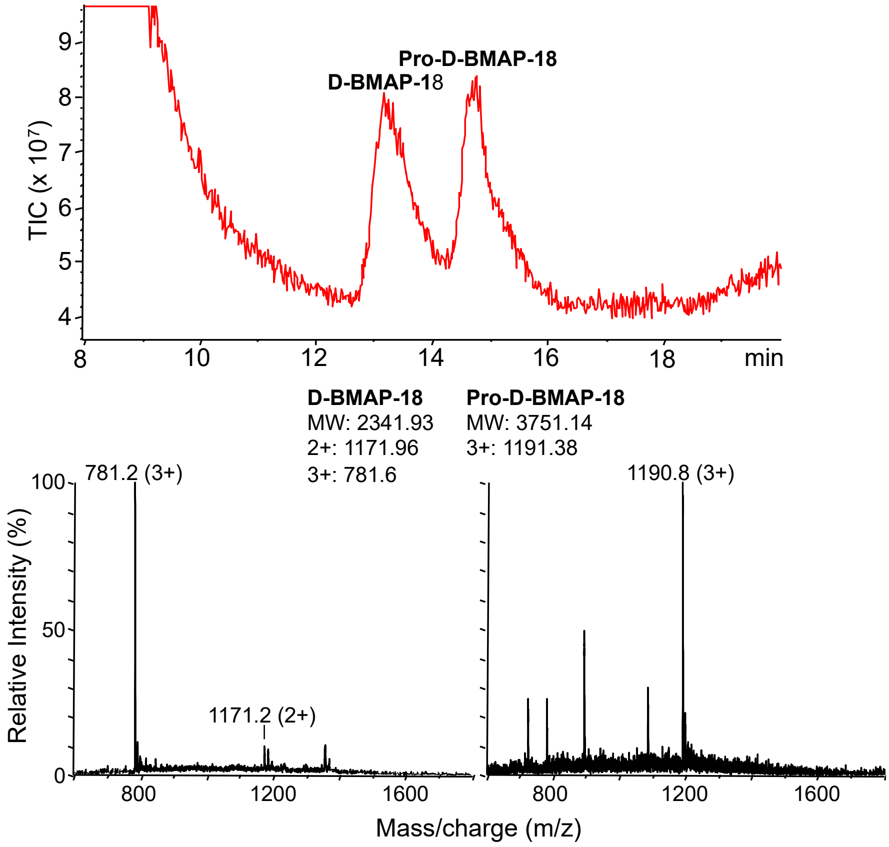

Figure S1. HPLC-MS spectra of the *D*-BMAP18 released after 1h-incubation of the Pro-*D*-BMAP18 with elastase (molar ratio1:100). The expected and observed MW of *D*-BMAP18 and Pro-*D*-BMAP18 in the MS analysis are reported in the right panel.

The capability of the activated peptide to eradicate a pre-formed biofilm was evaluated against the PA01, RP73, PA08 *P. aeruginosa* strains. The treatment with different concentrations of the pro-form did not alter biofilm vitality compared to the untreated control. In the presence of elastase the *D*-BMAP18 released by the pro-form has an antibiofilm activity equal to that-one of the free peptide. This result suggests that the cleavage by elastase enable the release of a *D*-BMAP18 which is fully active.

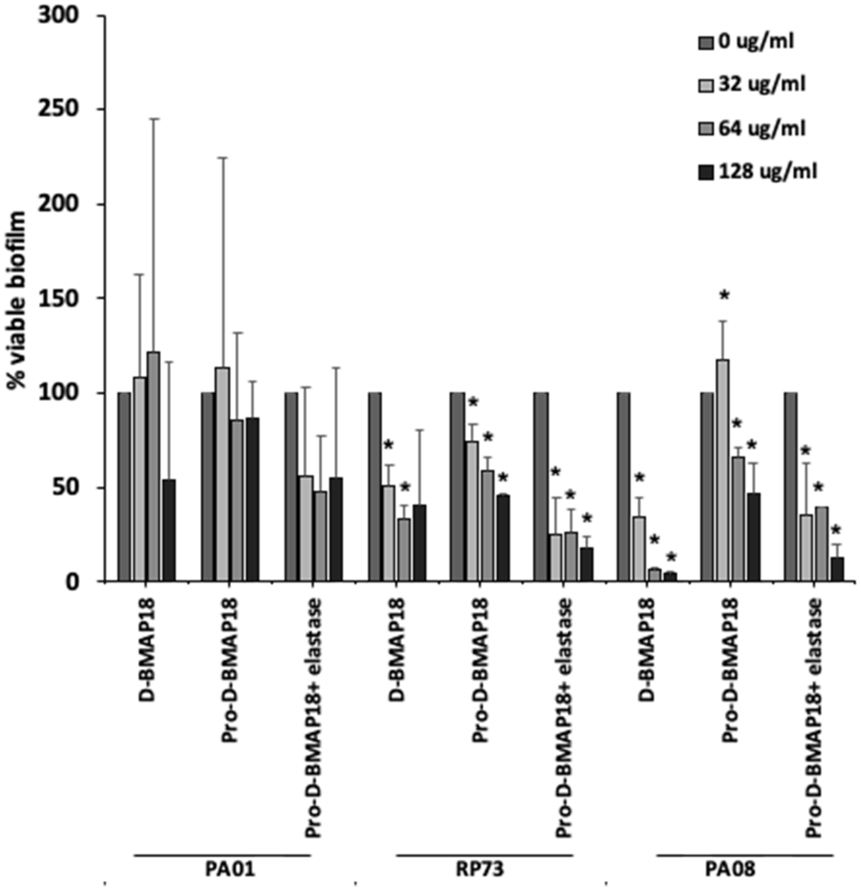

Figure S2. Eradication activity evaluated by a MTT assay. Percentage of viable biofilm after 24h of treatment at different concentrations of Pro-, *D*- and Pro-*D*-BMAP18 incubated with elastase are shown. Biofilm eradication assay was performed against the P. aeruginosa strains PA01, PA08 and RP73. After a 24h-treatment with the peptides, an MTT assay was done as reported in Mardirossian et al.[22]. The results are the average of three independent experiments in internal triplicate.* $p< 0.05$. Student t test of the treatments against the untreated control.
